# Venezuelan Equine Encephalitis Virus Activity in the Gulf Coast Region of Mexico, 2003–2010

**DOI:** 10.1371/journal.pntd.0001875

**Published:** 2012-11-01

**Authors:** A. Paige Adams, Roberto Navarro-Lopez, Francisco J. Ramirez-Aguilar, Irene Lopez-Gonzalez, Grace Leal, Jose M. Flores-Mayorga, Amelia P. A. Travassos da Rosa, Kali D. Saxton-Shaw, Amber J. Singh, Erin M. Borland, Ann M. Powers, Robert B. Tesh, Scott C. Weaver, Jose G. Estrada-Franco

**Affiliations:** 1 Institute for Human Infections and Immunity, Center for Tropical Diseases, and Department of Pathology, University of Texas Medical Branch, Galveston, Texas, United States of America; 2 Comisión México-Estados Unidos para la Prevención de la Fiebre Aftosa y Otras Enfermedades Exóticas de los Animales (CPA), Secretaría de Agricultura, Ganadería, Desarrollo Rural, Pesca y Alimentación (SAGARPA), Tuxtla Gutierrez, México; 3 Laboratorio de Enfermedades Emergentes y Zoonóticas, Facultad de Ciencias Químicas - Campus IV, Universidad Autónoma de Chiapas, Tapachula, Chiapas, México; 4 Centers for Disease Control and Prevention, Fort Collins, Colorado, United States of America; 5 Centro de Investigación y Estudios Avanzados en Salud Animal, Facultad de Medicina Veterinaria y Zootecnia, Universidad Autónoma del Estado de México, Toluca, México; USAMRIID, United States of America

## Abstract

Venezuelan equine encephalitis virus (VEEV) has been the causative agent for sporadic epidemics and equine epizootics throughout the Americas since the 1930s. In 1969, an outbreak of Venezuelan equine encephalitis (VEE) spread rapidly from Guatemala and through the Gulf Coast region of Mexico, reaching Texas in 1971. Since this outbreak, there have been very few studies to determine the northward extent of endemic VEEV in this region. This study reports the findings of serologic surveillance in the Gulf Coast region of Mexico from 2003–2010. Phylogenetic analysis was also performed on viral isolates from this region to determine whether there have been substantial genetic changes in VEEV since the 1960s. Based on the findings of this study, the Gulf Coast lineage of subtype IE VEEV continues to actively circulate in this region of Mexico and appears to be responsible for infection of humans and animals throughout this region, including the northern State of Tamaulipas, which borders Texas.

## Author Summary

Venezuelan equine encephalitis virus (VEEV) has been responsible for hundreds of thousands of human and equine cases of severe disease in the Americas. In 1969, an outbreak of Venezuelan equine encephalitis (VEE) spread rapidly from Guatemala and through the Gulf Coast region of Mexico, reaching Texas in 1971. Since this outbreak, there has been very little done to understand the ecology of VEEV in this region. Here, we present that the results of recent field studies that focus on confirming the continued existence of enzootic VEEV in the Gulf Coast region of Mexico. We performed serological analyses of sera collected between 2003 and 2010 from humans, cattle, horses, and dogs in various regions along the Gulf Coast of Mexico, and these data were complemented by wildcaught rodent serosurveys. Additionally, phylogenetic analyses were performed on VEEV isolates from this region to determine whether there have been substantial genetic changes in these viruses since the 1960s.

## Introduction

Since the first isolation and identification of Venezuelan equine encephalitis virus (VEEV) in the 1930s, outbreaks of Venezuelan equine encephalitis (VEE) have been periodically recognized as important occurrences of human and equine disease in the Americas [1], [2]. Major epidemics/epizootics during the 1970s and 1990s indicate a continued human and animal health threat of VEEV infection throughout the Americas, including the U.S. [3]–[5]. In the 1990s, outbreaks of VEE in Venezuela and Colombia as well as southern Mexico underscore the continued risk of VEE emergence [6]–[8].

In Mexico, the earliest recorded evidence of VEEV circulation in the Gulf Coast region came from human sera collected in Tlacotalpan in the State of Veracruz in 1961, where 7 individuals tested seropositive for VEEV by hemagglutination inhibition (HI) assays [9]. Beginning in 1962, VEEV was found to be circulating in the southern Gulf Coast region of Mexico, where deaths, neurological disease, and seropositive individuals were associated with a VEE outbreak in Champotón, Campeche [10]. Five deaths were recorded (38% case-fatality rate) and at least 3 surviving individuals developed neurological sequelae. Further studies carried out between 1962–1964 in the southeastern areas of the Gulf Coast, including the States of Quintana Roo, Yucatan, Tabasco and Veracruz, identified VEEV-seropositive individuals (3% seroprevalence) among the 770 individuals tested [9]. However, no clinical cases were documented during these studies.

The first VEEV subtype IE isolates from Mexico were obtained by Scherer et al. from sentinel hamsters and mosquito pools in 1963 in the Sontecomapan region of the State of Veracruz [11]. These studies noted the constant enzootic circulation of the subtype IE VEEVs in several lowland coastal habitats in the Gulf Coast region and suggested the potential threat of VEEV expansion mediated by the north-south movement of people and domestic animals in these areas. In 1965, a human fatality associated with subtype IE VEEV was reported in the village of Jaltipan, Veracruz [12]. Later, in 1966, an equine epizootic attributed to subtype IE VEEV took place in the Tampico region of the northeastern State of Tamaulipas, which borders Texas. During this epizootic, approximately 1,000 equids developed encephalitis and 300 deaths were recorded [13].

A VEE outbreak in 1969–72, caused by an epizootic strain of subtype IAB VEEV spreading northward from Guatemala, affected several coastal regions of Mexico and the U.S. [5]. In September, 1970, this epidemic/epizootic was first reported in the State of Veracruz in the San Andres Tuxtla region, and then, it continued to expand northward during the summer of 1971 through the Tuxpan-Tamiahua region of the State of Veracruz. A few days later, the outbreak spread to the municipality of Soto La Marina in the State of Tamaulipas, about 200 km (124 miles) from the U.S. border, before being reported in southern Texas, where approximately 1,500 horses died and several hundred human cases were documented [5], [14].

Immediately following the 1969–72 outbreak, there were no reported attempts to study VEEV circulation in Mexico. The last documented activity of VEEV in the Gulf Coast region of Mexico occurred in 1991 in the village of Paraiso in the State of Tabasco [15]. Two children (5- and 7-years old) seroconverted, implying recent VEEV exposures. Further surveillance in this village revealed an overall seroprevalence of 19.3% in the 394 individuals sampled (all ages). Additionally, subtype IE VEEV was isolated from mosquito pools collected from this area; however, the mosquito species were not reported [15]. Two years later, on the Pacific Coast of Mexico, a VEE epizootic occurred in the State of Chiapas in 1993, and then in the State of Oaxaca in 1996 [8], [15]. These outbreaks were attributed in part to the adaptation of the etiologic subtype IE strains to the mosquito vector, *Aedes* (*Ochlerotatus*) *taeniorhynchus* [16]. Subsequently, both endemic and enzootic VEEV circulation has been periodically documented in this region [JGEF and SCW, unpublished data].

Despite recent efforts to understand the ecology of VEEV in the Pacific Coast of Mexico, little has been done on the Gulf Coast since the 1960s. Here, we present that the results of field studies that focus on confirming the continued existence of enzootic VEEV in the Gulf Coast region of Mexico. This research was designed to expand our knowledge of the ecology and epidemiology of VEEV, primarily focused on the State of Veracruz, and to estimate the potential of VEEV to spread northward into regions near the Mexico-U.S. border, as occurred in 1971. Between 2003 and 2010, we performed serological surveillance studies of humans, cattle, horses, and dogs in various regions along the Gulf Coast of Mexico. These data were complemented by wild-caught rodent serosurveys to identify putative reservoir hosts. Additionally, phylogenetic analyses were performed on VEEV isolates from this region to determine whether there have been substantial genetic changes in these viruses since the 1960s, including the potential introduction of the epizootic Pacific Coast lineage of subtype IE VEEV into this area.

## Materials and Methods

### Ethics statement

For the human serosurveillance studies, a written informed consent was obtained from all subjects. For individuals less than 18 years of age, a written consent was obtained from a parent or legal guardian. For the animal surveys, all animal owners provided consent to have their animals involved in the study. The study in Mexico was also conducted under permit no. SGPA/DGVS/03858, issued to Dr. J.G. Estrada-Franco, by the Secretaría del Medio Ambiente Y Recursos Naturales de Mexico (SEMARNAT), and any animal work at the University of Texas Medical Branch (UTMB) was performed in strict accordance with the recommendations in the *Guide for the Care and Use of Laboratory Animals* of the National Research Council. The animal protocol was approved by the Institutional Animal Care and Use Committee at the UTMB. For the entomological surveys, all owners of private property gave permission for the study to be conducted on their land.

### Study areas

In 2003–2004, we performed serosurveys of equids in selected areas of the States of Tamaulipas, Veracruz, and Tabasco, as previously described [17]. In 2008–2010, a more focused study was performed in the State of Veracruz, where we tested for VEEV antibodies in a variety of vertebrates, including equids, bovids, canids, rodents and humans. We also isolated and sequenced viruses from sentinel hamsters and mosquitoes in this area (Table 1).

**Table 1:** VEE complex virus strains used in phylogenetic analysis.

Abbreviation^a^	Strain	Location	Date (yr or mo-yr^b^)	Source^c^	Passage history^d^	GenBank accession no.
IAB_TR43	Trinidad donkey	Trinidad	1943	Donkey	gp1, v6	LO1442
IAB_TX71	71–180	Texas	1971	Horse	sm1, h1, v1, C6/36-1	AF069903
IC_VE63	P676	Miranda State, Venezuela	8-1963	*A. triannulatus*	sm4, BHK1	AF375051
IC_VE95	3908	Sinamaica, Zulia State, Venezuela	9-1995	Human	C6/36-1	U55350
IC_VE92	243937	Trujillo State, Venezuela	12-1992	Horse	v1	AF004459
IC_VE95	6119	Falcon State, Venezuela	5-1995	Human	v1, BHK1	U55347
IC_VE93	SH3	Trujillo State, Venezuela	1-1993	Human	v1	U55360
ID_PA61	3880	Cañito, Panama	4-1961	Human	sm1, v8	L00930
ID_CO69	CoAn9004	Tumaco, Colombia	1969	Hamster	sm3, v1	GU085856
ID_VE81	66637	Sinamaica, Zulia State, Venezuela	11-1981	Hamster	v1, sm1	AF004458
ID_CO83	83U434	Norte de Santander Department, Colombia	6-1983	Hamster	cec1, v1	U55362
ID_VE97	MAC10	Miranda State, Venezuela	5-1997	Hamster	v2	GU085852
ID_VE97	ZPC738	Zulia State, Venezuela	9-1997	Hamster	BHK1	AF100566
ID_PA98	8131	Iquitos, Peru	1998	Human	BHK1	DQ390224
ID_PE06	FMD749	Puerto Maldonado, Madre de Dios, Peru	1-2006	Human	v1	GU085857
ID_BO06	FVB204	Eterazama, Cochabamba, Bolivia	4-2006	Human	v1	GU085854
ID_BO06	FVB200	Eterazama, Cochabamba, Bolivia	3-2006	Human	v1	GU085853
ID_PE07	FMD1070	Puerto Maldonado, Madre de Dios, Peru	2-2007	Human	v1	GU085858
ID_BO07	FVB258	Eterazama, Cochabamba, Bolivia	2-2007	Human	v1	GU085855
IE_PA62	MenaII	Almirante, Panama	1962	Human	sm3, v1	AF075252
IE_GU68	68U201	La Avellana, Santa Rosa Department, Guatemala^e^	1968	Hamster	sm3, v2, BHK1	U34999
IE_GU73	73U151	La Avellana, Santa Rosa Department, Guatemala^e^	1973	Hamster	?, v1	JQ859940
IE_GU77	77U208	La Avellana, Santa Rosa Department, Guatemala^e^	1977	Hamster	?, v1	JQ859941
IE_GU80	80U76	La Avellana, Santa Rosa Department, Guatemala^e^	1980	Hamster	C6/36-1	AF448539
IE_MX96	OAX131	Oaxaca State, Mexico	1996	Horse	sm1, RK1, C6/36-1	AF448536
IE_MX96	OAX142	Oaxaca State, Mexico	1996	Horse	sm1, RK1, C6/36-1	AF448538
IE_MX01	22	Las Coaches, Chiapas State, Mexico	7-2001	Hamster	BHK1	AY823299
IE_MX63	63U16	Veracruz, Mexico	1963	Hamster	sm1, C6/36-1	GU085860
IE_MX63	63Z1	Veracruz, Mexico	8-1963	Human	sm1, v1	JQ859933
IE_MX65	65U206	Sontecomapan, Veracruz State, Mexico	1965	Hamster	sm1, v1	JQ859934
IE_MX66	66U11	Minatitlan, Veracruz State, Mexico	1966	Hamster	cec1, v1	JQ859935
IE_MX67	67U222	Minatitlan, Veracruz State, Mexico	1967	Hamster	?, v2	JQ859936
IE_HON67	67U225	Puerto Cortez, Honduras	1967	Hamster	sm1, v1	JQ859937
IE_MX69	69U315	Sontecomapan, Veracruz State, Mexico	1969	Hamster	sm1, v1	JQ859938
IE_GU70	70U74	Puerto Barrios, Izabel Department, Guatemala^e^	8-1970	Hamster	?, v1	JQ859939
IE_GU79	79U13	Izabel Department, Guatemala^e^	1979	Hamster	?, v1	JQ859942
IE_MX08	H50	E. Coachapa, Minatitlan, Veracruz State, Mexico	7-2008	Hamster	v1	JQ859943
IE_MX08	H51	E. Coachapa, Minatitlan, Veracruz State, Mexico	7-2008	Hamster	v1	JQ859944
IE_MX08	H52	E. Coachapa, Minatitlan, Veracruz State, Mexico	7-2008	Hamster	v1	JQ859945
IE_MX08	H53	Tacoteno, Minatitlan, Veracruz State, Mexico	7-2008	Hamster	v1	JQ859946
IE_MX08	H54	Tacoteno, Minatitlan, Veracruz State, Mexico	7-2008	Hamster	v1	JQ859947
IE_MX08	H55	Tacoteno, Minatitlan, Veracruz State, Mexico	7-2008	Hamster	v1	JQ859948
IE_MX08	H56	Tacoteno, Minatitlan, Veracruz State, Mexico	7-2008	Hamster	v1	GU085859
IE_MX08	H58	Tacoteno, Minatitlan, Veracruz State, Mexico	7-2008	Hamster	v1	JQ859949
IE_MX09	H60	Tacoteno, Minatitlan, Veracruz State, Mexico	9-2009	Hamster	v1	JQ859950
IE_MX10	H91	Tacoteno, Minatitlan, Veracruz State, Mexico	8-2010	Hamster	v1	JQ859951
IE_MX10	H94	Tacoteno, Minatitlan, Veracruz State, Mexico	8-2010	Hamster	v1	JQ859952
IE_MX10	H95	Tacoteno, Minatitlan, Veracruz State, Mexico	8-2010	Hamster	v1	JQ859953
IE_MX09	M48	Tacoteno, Minatitlan, Veracruz State, Mexico	9-2009	*Cx. nigripalpus*	v1	JQ859954
IE_MX09	M49	Tacoteno, Minatitlan, Veracruz State, Mexico	9-2009	*Cq. nigricans*	v1	JQ859955
IE_MX09	M50	Tacoteno, Minatitlan, Veracruz State, Mexico	9-2009	*M. titillans*	v1	JQ859956
IE_MX09	M51	Tacoteno, Minatitlan, Veracruz State, Mexico	9-2009	*Cx. nigripalpus*	v1	JQ859957
IE_MX09	M64	Tacoteno, Minatitlan, Veracruz State, Mexico	9-2009	*Cx. taeniopus*	v1	JQ859958
II_FL63-EVEV	Fe37c	Everglades, Florida, USA	1963	*Cx. (Mel.)* spp.	sm4, v6	AF075251
IIIA_BR54-MUCV	BeAn8	Belem, Brazil	12-1954	Monkey	p8	AF075253

a Venezuelan equine encephalitis complex viruses; subtype precedes underscore; EVEV, Everglades virus; MUCV, Mucambo virus.

b Month of isolation is provided, if available.

c Hamster refers to sentinel hamster.

d sm, suckling mouse; v, Vero cell culture; p, unknown passage source; gp, guinea pig; ch, chicken embryo; m, mosquito; C6/36, C6/36 *Aedes albopictus* cell culture; dec, duck embryo cell culture; rd, human embryonal rhabdomyosarcoma cell culture; BHK, baby hamster kidney cell culture; CEC, chick embryo cell culture; h, horse; RK, RK-12 cell culture; ?, unknown passage source or number.

e Puerto Barrios, Guatemala is on the Atlantic coast, and La Avellana, Guatemala is on the Pacific coast.

#### Localities sampled in the State of Tamaulipas (2003–2004)

The majority of horse sera collected in the State of Tamaulipas came from an experimental livestock station associated with the Instituto Nacional de Investigaciones Forestales, Agrícolas y Pecuarias (INIFAP), a Mexican agricultural research institute, in the Aldama region (see Table 2 for grid coordinates). This is a low, humid, semiarid coastal municipality devoted mainly to fishing and livestock production. Vegetation in this area includes marsh grasses, brushes, and weeds. Three rivers flood the coastal plains year-round, including the Carrizal, Barbarena, and Tigre. At the Mexico-Texas border, within the framework of the West Nile virus surveillance activities of the Mexican Agriculture Ministry, we also sampled horses in ranches in three border towns adjacent to the Texas counties of Cameron and Hidalgo, including Camargo, Diaz Ordaz, and Matamoros. Vegetation in these regions is typical for subtropical Gulf prairies with bluestems and grama grasses predominating during mild winters and hot summers. The border towns are located within the Rio Bravo basin. Finally, equine samples were also obtained from the municipality of Jaumave, located near the capital city of Victoria in the mountainous region of the State of Tamaulipas.

**Table 2:** Equine seroprevalence for Venezuelan equine encephalitis virus (VEEV) in the Mexican Gulf States of Tamaulipas, Veracruz, and Tabasco, 2003–2004.

State	Municipality	No. of equids tested	No. of seropositive equids^a^^,^ b ^,^ c	Prevalence	Coordinates
Tamaulipas	Camargo	10	1^a^^,^ b	10%	N24°52′07.02″;W97°49′22.77″
	Diaz Ordaz	10	1^a^^,^ b	10%	N25°41′24.58″;W97°26′50.86″
	Matamoros	9	2^a^^,^ b ^,^ c	22%	N25°49′15.54″;W97°28′11.45″
	Jaumave	9	3^b^^,^ c	33%	N23°24′34.10″;W99°21′55.52″
	Aldama	91	15^b^^,^ c	16%	N22°54′39.85″;W98°02′23.11″
	Total	**129**	**22**		
	Average			**17%**	
Veracruz	Hidalgotitlan	7	4^b^^,^ c	57%	N17°46′02.96″;W94°38′34.83″
	Minatitlan	15	15^b^^,^ c	100%	N17°57′23.85″;W94°32′16.93″
	Texistepec	5	3^b^^,^ c	60%	N17°53′29.68″;W94°49′16.46″
	A. R. Cabada	6	6^b^^,^ c	100%	N18°35′33.83″;W95°26′57.76″
	Las Choapas	6	6^b^^,^ c	100%	N17°54′39.61″;W94°06′04.18″
	Tlacotalpan	10	7^b^^,^ c	70%	N18°36′59.25″;W95°39′21.32″
	Cosamaloapan	17	13^b^^,^ c	76%	N18°22′27.61″;W95°48′08.13″
	J Carranza	10	2^b^^,^ c	20%	N17°26′11.32″;W95°01′30.98″
	Panuco	3	1^b^^,^ c	33%	N22°03′17.69″;W98°11′35.44″
	Total	**79**	**57**		
	Average			**72%**	
Tabasco	Tenosique	45	26^b^	58%	N17°28′15″;W91°25′35″
	Balancan	40	25^b^	63%	N17°48′25″;W91°32′08″
	Jonuta	43	11^b^^,^ c	25%	N18°05′44.88″;W92°08′12.24″
	Centla	40	8^b^^,^ c	20%	N18°00′51.03″;W92°53′43.97″
	Jalpa	40	15^b^^,^ c	38%	N18°10′20.65″;W93°03′12.39″
	Centro	178	61^b^^,^ c	34%	N17°57′11.85″;W92°54′21.09″
	Macuspana	40	40^b^	100%	N17°45′34″;W92°35′52″
	Comalcalco	40	39^b^	98%	N18°16′16″;W93°13′30″
	Paraiso	40	40^b^	100%	N18°23′44″:W93°12′44″
	Total	**506**	**265**		
	Average			**52%**	

a Results based on IgM and IgG ELISA; this assay was only performed on equine sera collected from the State of Tamaulipas.

b Results based on hemaglutination-inhibition (HI) assay.

c Results based on plaque reduction neutralization test (PRNT); titers ≥1∶20 were considered positive.

#### Localities sampled in the State of Veracruz (2003–2004)

In the State of Veracruz, we conducted an equine serosurvey in the Papaloapan-Coatzacoalcos river basin, including the towns of Hidalgotitlan, Minatitlan, Texistepec, A. R. Cabada, Las Choapas, Tlacotalpan, Cosamaloapan, and Jesus Carranza (see Table 2 for grid coordinates). Overall, the region is an area of savannah and extensive tropical rain forests with freshwater habitats, particularly swamps and marshes near the Coatzacoalcos River. Finally, samples were also collected in the area of Panuco in northern region of the State of Veracruz and near the port city of Tampico, which has a tropical rain forest coastal habitat.

#### Localities sampled in the State of Tabasco (2003–2004)

In the State of Tabasco, 9 municipalities were sampled for equine serological surveillance, some near the Guatemala border in the south, including Tenosique and Balancan (see Table 2 for grid coordinates). Other municipalities included those in the state's central plains, including Jonuta, Centla, Jalpa, Centro, and Macuspana as well as the coastal and mangrove areas of Comalcalco and Paraiso. This region is largely composed of lowland humid tropical grasslands and forests as well as wetlands along the coastal habitats, which are irrigated by the Usumacinta and Grijalva River systems and their tributaries.

#### Localities sampled in the State of Veracruz (2008–2010)

For the studies carried out in the summers of 2008 to 2010, our focus was initially in the southeastern areas of the State of Veracruz in the Coatzacoalcos River basin and the nearby city of Minatitlan, a metropolitan area with at least 300,000 residents that is part of an industrial corridor closely linked to the oil industry. Later, two “ejidos” or communal lands were studied northeast [“Ejido Coachapa” (N17°53′25.50″; W94°33′33.30″)] or south [“Ejido Tacoteno” (N18°00′27.02″; W94°31′00.99″)] of Minatitlan, each a few hundred meters from urban areas. These two areas included wetlands with swamps and patches of grasslands devoted to raising cattle. These habitats included canopies of fruit trees (mango, coconut, and papaya), surrounded by flooded swamps with emergent aquatic vegetation, and were irrigated by the Coatzacoalcos River and its tributaries.

In 2010, bovine and equine serosurveys were also carried out in 19 farms in the municipalities of Tuxpan, Tamiahua, Coxquihui, Alamo, Tihuatlán, and Tecolutla of the State of Veracruz (see Table 3 for grid coordinates). These municipalities were located in the northern region of the State of Veracruz as far south as N20°11′; W97°35′ (Coxquihui) and as far north as N21°17′; W97°27′ (Tamiahua). The farms were located in semi-humid areas with tropical forests and perennial plants and included the predominantly marshy conditions of the coast.

**Table 3:** Equine and bovine seroprevalence in the northern region of the State of Veracruz, 2010.

			Bovine			Equine		
Municipality	Locality	Coordinates	No. of bovids tested	No. of seropositive bovids^a^	Prevalence	No. of equids tested	No. of seropositive equids^a^	Prevalence
Tuxpan	El Cuervo	N20°54′34″;W97°30′53″	18	6	33%	-	-	-
	El Taconazo	N20°54′31″;W97°17′16″	-	-	-	1	1	100%
	Los Angeles	N20°57′24″;W97°33′34″	-	-	-	3	2	67%
	Santa Monica	N20°48′55″;W97°19′39″	-	-	-	4	2	50%
	U. Veracruzana	N20°57′05″;W97°27′27″	-	-	-	3	2	67%
	Las Minas	N21°06′00″;W97°27′56″	18	5	28%	-	-	-
	Tampiquillo	N20°57′55″;W97°32′29″	-	-	-	2	2	100%
		Total	**36**	**11**		**13**	**9**	
		Average			**31%**			**69%**
Tamiahua	Temapache	N21°15′40″;W97°30′29″	15	2	13%	-	-	-
	Barra	N21°19′10″;W97°25′25″	4	3	75%	4	3	75%
	Tamiahua	N21°11′53″;W97°27′49″	10	10	100%	-	-	-
	El Carmen	N21°28′18″:W97°20′51″	29	5	17%	-	-	-
		Total	**58**	**20**		**4**	**3**	
		Average			**34%**			**75%**
Coxquihui	Coxquihui	N20°10′37″;W97°24′29″	3	1	33%	-	-	-
		Total	**3**	**1**				
		Average			**33%**			
Alamo	Las Palmas	N20°55′00″;W97°41′00″	-	-		11	3	27%
	Alamo	N21°06′00″;W97°43′49″	13	3	23%	-	-	-
		Total	**13**	**3**		**11**	**3**	
		Average			**23%**			**27%**
Tihuatlan	La Limonaria	N20°39′54″;W97°26′20″	5	0	-	5	3	60%
	Terrero	N20°47′49″;W97°32′42″	11	3	27%	1	1	100%
		Total	**16**	**3**		**6**	**4**	
		Average			**18%**			**66%**
Tecolutla	Chichicatzapan	N20°21′43″;W97°06′24″	-	-	-	5	4	80%
		Total				**5**	**4**	
		Average						**80%**

a Results based on hemaglutination-inhibition (HI) assay and the plaque reduction neutralization test (PRNT).

### Equine and bovine serosurveys

Equids (≥1.5 years old) were bled from the jugular vein, while bovids (6–24 months old) were bled from the median coccygeal vein at the base of the tail. From each animal, 5 ml of blood was collected into vacutainer tubes (Beckton Dickinson, Franklin Lake, NJ), centrifuged for serum separation, and stored at 4°C before being transported to the laboratories at the Centers for Disease Control and Prevention (CDC, Ft. Collins, CO) and UTMB (Galveston, TX) for further processing.

We conducted 3 separate equine and/or bovine serosurveys between 2003 and 2010. The first one occurred in 2003–2004 in the States of Tabasco, Veracruz and Tamaulipas. Of the total number of equids sampled (n = 714), 18% (129/714) originated from 5 municipalities in the State of Tamaulipas, 11% (79/714) originated from 9 municipalities in the State of Veracruz, and 71% (506/714) originated from 9 municipalities in the State of Tabasco. The second serosurvey was conducted in 2008–2009 in Minatitlan in the State of Veracruz in previously identified areas of VEEV circulation. Here, we tested the sera of 81 bovids and 27 equids. The third serosurvey was carried out during the summer of 2010 in 6 municipalities of the northern region of the State of Veracruz, including municipalities near the State of Tamaulipas. For this serosurvey, 126 cattle and 53 horses were tested to estimate the northern geographical extension of VEEV within the State of Veracruz.

Sera collected from horses and cattle in 2003–2010 in the States of Veracruz and Tabasco were tested in almost all cases by both plaque reduction neutralization test (PRNT) and HI assays. The one exception was horse sera collected in the State of Tamaulipas in 2003–2004, in which IgM/IgG enzyme-linked immunosorbent assays (ELISAs) were also performed.

### Canine serosurveys

In 2008–2009, companion dogs (≥6 months old) were serosurveyed in areas surrounding the Ejido Coachapa in the municipality of Minatitlan in the State of Veracruz. Blood (5 ml) was obtained via venipuncture of the cephalic vein and processed as described above. Sera were tested by both PRNT and HI assays.

### Rodent serosurveys

In 2008–2009, wild-caught rodents were collected with Sherman live traps (H.B. Sherman Traps, Inc., Tallahassee, FL) in the ejidos of Coachapa and Tacoteno in the municipality of Minatitlan. Traps were placed next to swampy pastures and streams of farms where we had also observed sentinel hamster mortalities and/or positive equine and bovine serology for VEEV. Trapped rodents were anesthetized and bled by cardiac puncture; approximately 1 ml of blood was transferred to vacutainer tubes (Becton Dickinson, Franklin Lake, NJ) for serum separation and stored at −20°C until further processing. Smaller rodents were bled from the retro-orbital sinus with heparinized capillary tubes, from which plasma was collected after centrifugation of the blood and stored at −20°C until processed. Sera were tested by both PRNT and HI assays.

### Human serosurveys

Between October 2008 and April 2009, sera from 234 individuals of all ages were obtained by the Veracruz Health Services as part of an ongoing dengue surveillance program. These sera were collected from acute febrile patients from 44 municipalities broadly representing the State of Veracruz. This included Minatitlan, a municipality where we had detected VEEV circulation based on animal serology and virus isolation. Sera were tested in all cases by PRNT, and in most cases, by IgG ELISAs. PRNT- and IgG-negative serum was tested for IgM antibodies to VEEV. Serum samples from two municipalities (Minatitlan, Villa Acula) were also tested by HI assay.

### IgM ELISA

The IgM capture ELISA was performed essentially as previously described [18]. Briefly, 96-well Immulon II HB plates (Dynatech Industries, Chantilly, VA) were coated with 75 µl of either goat anti-horse or goat anti-human IgM (Kirkegaard and Perry Laboratories, Gaithersburg, MD) in carbonate/bicarbonate buffer. Fifty microliters per well of 1∶400 wash buffer-diluted sample or control sera were added to the wells and allowed to incubate for 1 h at 37°C in a humidified chamber. Either viral or uninfected control antigen that was produced from cell lysates was diluted in wash buffer and 50 µl/well was added in triplicate to the appropriate wells where they were allowed to incubate overnight at 4°C. A horseradish peroxidase-conjugated monoclonal antibody (MAb) (Jackson Immunological Laboratories, West Grove, PA) was then used as a detector antibody. Calculations of P/N values were performed by following the guidelines of previous studies [18]. For a specimen to be considered IgM positive to the test virus, the P/N ratio (OD reading of sample on viral antigen/OD reading of normal control sera on viral antigen) must be ≥3 and the value of P for the test specimen must be greater than or equal to twice the mean OD of the test specimen reacted on normal antigen.

### IgG ELISA

To detect the presence of VEEV IgG in serum samples, a previously described monoclonal antibody-based capture ELISA protocol was followed [19]. Briefly, a 96-well Immulon II HB plate was coated with 75 µl/well of the broadly cross-reactive alphavirus MAb, which was diluted in carbonate/bicarbonate buffer. Blocking buffer [phosphate buffered saline (PBS) with 0.5% Tween 20 and 5% nonfat dry milk] was added and allowed to incubate as described previously. Either viral or uninfected control antigen that was produced from cell lysates was diluted in wash buffer and added to the appropriate wells and allowed to bind the MAb. Either sample sera or control sera (positive and negative) diluted 1∶400 in wash buffer were added to triplicate wells and allowed to incubate for 1 h at 37°C. Either goat anti-horse or goat anti-human IgG-alkaline phosphatase conjugated antibody was then added, and samples were read on a plate reader as directed.

### HI assays

For the HI assays, VEEV subtype IE-specific antibodies were detected using an antigen derived from subtype IE VEEV strain 68U201 (isolated in 1968 from the brain of a sentinel hamster near La Avellana, in the Pacific lowlands of southeastern Guatemala). The sera were also screened by HI for 3 other arboviruses known to circulate in Mexico: eastern equine encephalitis virus (EEEV; strain TenBroeck), West Nile virus (WNV; strain 385-99), and St. Louis encephalitis virus (SLV; strain TBH28). Briefly, 4–8 units of hemagglutinin antigen from each virus were reacted with heat-inactivated serially diluted serum, starting at a dilution of 1∶20, and failure to hemagglutinate goose erythrocytes was considered a positive result.

### PRNTs

PRNTs were performed as previously described [20]. Briefly, starting at a dilution of 1∶10, heat-inactivated serum samples were serially diluted in Dulbecco's modified eagles medium (DMEM) with 5% fetal bovine serum (FBS) and incubated with ∼100 plaque forming units (PFU) of VEEV subtype IE strain 68U201 at 37°C for 1 h. Confluent monolayers of African Green Monkey kidney (Vero) cells in 12-well plates were inoculated with 100 µl of the virus/serum mixture and incubated at 37°C for 1 h. A 0.4% agarose overlay was then applied to each well and incubated at 37°C for 48 h before being fixed in 10% formalin and stained with 20% methanol/0.25% crystal violet. Serum dilutions resulting in ≥80% reduction in virus plaque titers were considered positive, with titers reported as the reciprocal of the endpoint dilution. Sera collected from horses, cattle, and dogs in areas outside of known endemic regions (Toluca Valley near Mexico City, 2,650 meters altitude) were used as negative controls.

### Sentinel hamsters

Golden Syrian hamsters were used as bait in modified Trinidad mosquito traps to isolate VEEV from infected hamsters and transmitting mosquitoes [21]. Our approach needed to overcome the simultaneous circulation of Group C (including Nepuyo virus) and Patois viruses circulating in our field study areas in Minatitlan [JGEF and SCW, unpublished data]. Therefore, to reduce deaths associated with infections of both viruses, hamsters were first treated with antisera obtained from the ascitic fluid of mice previously infected with Nepuyo and Patois viruses. Fifteen hamsters received a subcutaneous injection of 0.2 ml of ascitic fluid diluted to 1% in PBS prior to field exposure in the Trinidad mosquito traps. The baited traps were placed on farms under the canopy of fruit trees and suspended about 1.5 meters above the ground. Hamsters were provided carrots, orange slices, sunflower seeds, and water. Hamsters that became moribund or died in the traps were dissected, and tissues (primarily brain, heart, and spleen) were placed immediately in cryovials and liquid nitrogen containers in the field.

### Mosquito collections

Mosquito collections occurred in June–July of 2008–2010, October of 2009, and February of 2010, and were primarily concentrated in the Tacoteno region and in other areas surrounding Minatitlan in the State of Veracruz. At least 12 mosquito traps were set out daily in a 10-day interval in an area of approximately 15 square meters. The traps were checked for mosquitoes early each morning, and mosquitoes were removed from the traps with mechanical aspirators and placed in cardboard containers. The mosquitoes were provided with a 10% sucrose solution until they were processed. The mosquitoes were sorted by species and placed into cryovials as pools with a maximum of 30 individuals/vial, and then mosquito pools were stored in liquid nitrogen for transportation to UTMB for viral isolation.

### Virus isolation from hamster tissues and mosquito pools

VEEV was isolated from 10% hamster heart tissue suspensions in DMEM supplemented with 10% FBS and penicillin-streptomycin. Tissues were homogenized in Safe-Lock tubes (Eppendorf, Hauppauge, NY) with 5 mm stainless steel balls 450 grade A and homogenized in a Qiagen TissueLyser (Retsch, Newton, PA) for 4 min at 26,000 oscillations per minute. After centrifugation, the supernatant was inoculated onto confluent monolayers of Vero cells and incubated at 37°C for 3–5 days or until cytopathic effects (CPE) were evident.

Mosquito pools from the traps in which a hamster became infected were assayed for infectious virus. Pools containing 1–30 mosquito specimens were triturated and processed using the same method as the hamster tissues. The triturated pool was then centrifuged at 10,000 rpm and 4°C for 5 min. One hundred microliters of the supernatant was added to a 12.5 cm^2^ flask containing a monolayer of Vero cells, incubated at 37°C for 1 h, and then, 5 ml of DMEM with 5% FBS was added to the flask. The cultures were monitored for CPE for the next 3–5 days.

Viruses isolated from hamster heart suspensions and mosquito pools were screened antigenically for the presence of alphaviruses using an indirect immunofluorescence assay (IFA) with monoclonal antibodies, as described previously [22].

### RNA isolation, RT-PCR, and sequencing

Viral RNA was isolated from hamster tissues and mosquito homogenates using the QIAamp® Viral RNA Mini kit (Qiagen, Valencia, CA) according to the manufacturer's instructions. cDNA synthesis and PCR amplification were performed in a one-step reaction using a Titan One-Tube RT-PCR kit (Roche Diagnostics, Indianapolis, IN) according to the manufacturer's protocol. A 2,500 nt amplicon was generated covering genomic nucleotide positions 7894–10395, which extended from the 3′ end of the capsid gene to the 5′ end of the E1 gene within the structural protein open reading frame. Table S1 lists the primers used for RT-PCR and sequencing. PCR amplicons were purified from agarose gels using the QIAquick Gel Extraction Kit (Qiagen) according to the manufacturer's instructions and sequenced directly using internal primers and an Applied Biosystems (Foster City, CA) Prism automated DNA sequencing. Nucleotide sequences derived in this study, including those from previous isolates from either our collection or the World Reference Center for Emerging Viruses and Arboviruses at UTMB, were submitted to GenBank under accession numbers JQ859933–JQ859958.

### Phylogenetic analyses

Sequences were aligned and compared to VEEV sequences available in the GenBank database (Table 1). Amino acid sequence alignments were performed using the ClustalW in SeaView version 4.2.12 [23], and the nucleotide sequences were aligned manually based on codon homology. A set of 55 sequences, which included reference strains for other VEEV subtypes, was trimmed to a minimum common length of 1,677 nt. Phylogenetic analyses were performed using the neighbor joining (NJ) and maximum parsimony algorithms implemented in the PAUP* version 4.0 software package [24], [25] as well as the maximum likelihood (ML) algorithm implemented in PhyML 3.0 (http://atgc.lirmm.fr/phyml) [26]. For NJ analysis, the HKY85 distance formula was used, and bootstrap analyses were performed with 1,000 replicates to place confidence values on the nodes within trees [27]. For ML analysis, the general time-reversible (GTR) model of nucleotide substitution was used for the data set, and the starting tree in the analysis was found using BIONJ (an improved version of the NJ algorithm) [28], which was followed by successive rounds of tree bisection reconstruction branch-swapping, identifying the ML substitutions model at each stage until the tree of highest likelihood was found. Bootstrapping was subsequently performed to assess the robustness of tree topologies using 1,000 replicate BIONJ trees under a ML substitution model.

## Results

### Seroprevalence of horses for VEEV in the States of Tamaulipas, Veracruz, and Tabasco, 2003–2004

**Figure 1:**
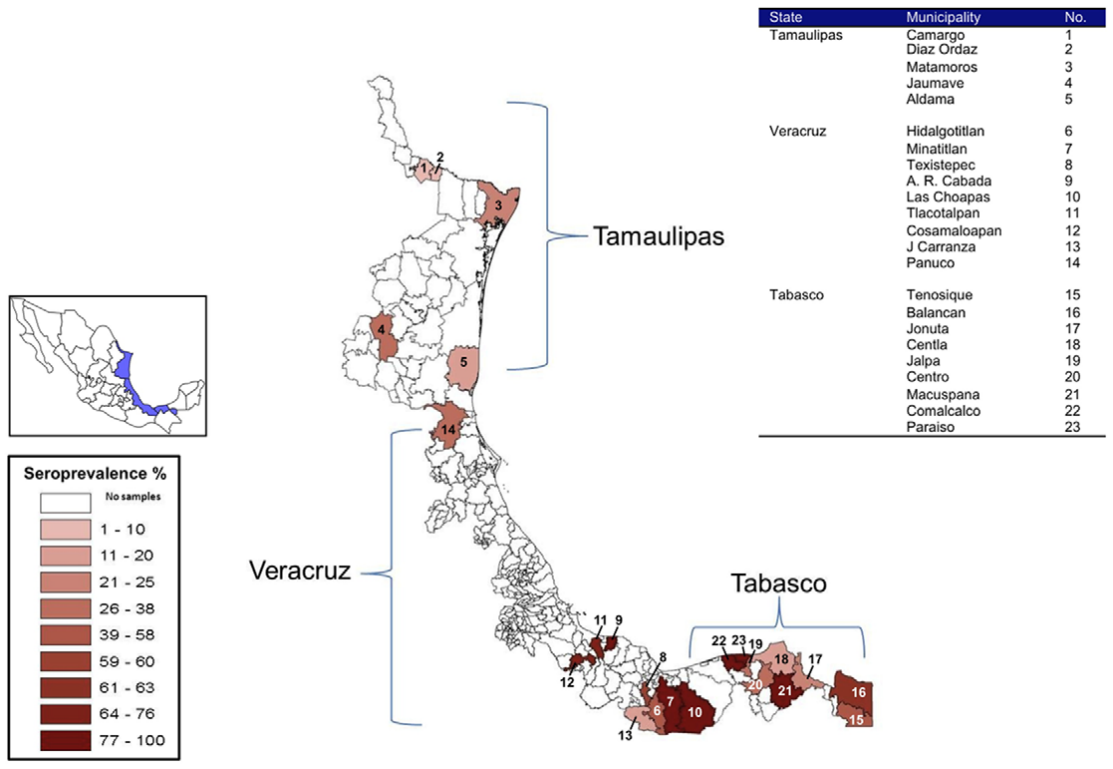
Geographical distribution and frequency of equids in the municipalities of three Gulf States of Mexico (Tamaulipas, Veracruz, Tabasco) with positive serology by ELISA, PRNT, and/or HI for Venezuelan equine encephalitis virus (VEEV), 2003–2004. Map inset shows the locations of the States of Tamaulipas, Veracruz, and Tabasco relative to the other states in Mexico. Table inset provides a location key for each municipality. Key inset shows the relative frequency (%) of positive samples based on color intensity.

A total of 714 equine sera collected in 2003–2004 from the Gulf Coast States of Tamaulipas (n = 129), Veracruz (n = 79), and Tabasco (n = 506), as part of a large West Nile virus epidemiology study conducted by the Mexican Ministry of Agriculture, was analyzed for VEEV antibodies [17]. Sera were tested in most cases by both PRNT and HI assays, and the results of these assays were consistently either positive or negative for VEEV. Horse sera collected in the State of Tamaulipas in 2003–2004 were also tested by ELISA, and the results of this assay were consistent with those of the PRNT and HI assays. Fig. 1 shows the geographic distribution and equine seropositivity in the municipalities of these states. None of the seropositive horses from this study were previously vaccinated against VEE. Further, compulsory VEE vaccination of horses with the live attenuated vaccine, TC-83, can only be carried out by the Agricultural Committees in the Pacific region of the State of Chiapas, and vaccination in other states is not permitted in the absence of an outbreak. Of 129 equids tested from the State of Tamaulipas, 22 (17% seroprevalence) had VEEV antibodies (Table 2). Four of the 22 seropositive horses (4–8 years old) were from 3 towns in the State of Tamaulipas (Camargo, Diaz Ordaz, and Matamoros) located near the U.S. border. Two horses from Matamoros were seropositive for VEEV by PRNT [1∶40] and IgG ELISA, while the other horses from Camargo (n = 1) and Diaz Ordaz (n = 1) were seropositive by both IgG and HI tests. Unfortunately, information regarding the travel histories of all 4 horses was lacking.

For the 91 horses that were tested in the municipality of Aldama, 15 were seropositive, 7 of which were located in a research station and had never been transported from this area since birth. It was also confirmed in medical records that these horses were never immunized against VEEV, supporting the possibility of local enzootic exposure. Based on these results, more direct studies would need to be conducted to confirm active transmission of VEEV in this region.

The equine serological data also suggest that VEEV-enzootic regions of the Gulf Coast included the States of Tabasco and Veracruz, particularly lowland areas (Fig. 1, Table 2). Of 79 equids tested in the State of Veracruz, the seroprevalence for VEEV was 72% (57/79). For the municipalities of Minatitlan, A. R. Cabada, and Las Choapas, the seroprevalence for VEEV was 100%. In the State of Tabasco, the seroprevalence for VEEV in equids was 52% (265/506). The southernmost areas, where VEEV-positive sera were detected, included the municipalities of Balancan and Tenosique in the State of Tabasco, which border Guatemala. In these municipalities, equine seroprevalence for VEEV was ≥58%. For the municipalities of Macuspana and Paraiso, the seroprevalence for VEEV was 100%.

### Seroprevalence of horses and cattle for VEEV in the State of Veracruz, 2008–2010

Based on the work conducted in 2003–2004, the State of Veracruz was the most active region for VEEV circulation with an overall equine seroprevalence of 72% (Table 2). Putative “hotspots” were identified in the southern part of this state, which guided more intensive serosurveys in 2008–2009. For this study, both equine and bovine serosurveys were conducted in and around the city of Minatitlan, in which the sera were tested in almost all cases by both PRNT and HI assays; the results of these assays were consistently either positive or negative for VEEV. Located northeast of the city, Ejido Tacoteno showed 100% seroprevalence in both equids (n = 7) and bovids (n = 10), and similar seroprevalence (n = 14 equids; n = 59 bovids) was found in Ejido Coachapa, which is located south of Minatitlan in the Coatzacoalcos river basin. These data also corroborated evidence from our earlier study suggesting that VEEV is enzootic in the Minatitlan region in the southern region of the State of Veracruz.

**Figure 2:**
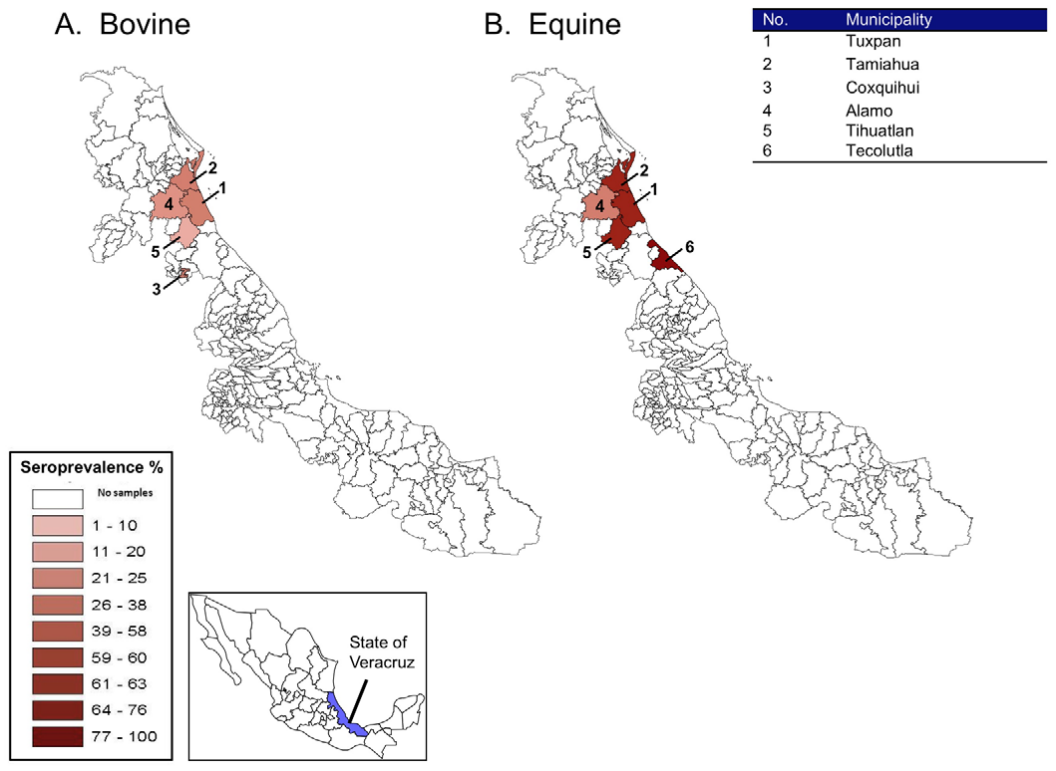
Geographical distribution and frequency of bovids and equids in the northern municipalities of the State of Veracruz with positive serology by ELISA and PRNT for Venezuelan equine encephalitis virus (VEEV), 2010. Maps show the distribution and seroprevalence for VEEV in bovids (A) and equids (B). Map inset shows the location of the State of Veracruz relative to the other states in Mexico. Table inset provides a location key for each municipality. Key inset shows the relative frequency (%) of positive samples for based on color intensity.

In 2010, equine and bovine serosurveys were also performed in the northern region of the State of Veracruz, involving 6 municipalities (Table 3; Fig. 2). As before, sera were tested in almost all cases by both PRNT and HI assays, and the results of these assays were consistently either positive or negative for VEEV. The overall seroprevalence for VEEV was 30% (38/126) in bovids and 59% (23/39) in equids. However, for the municipalities of Tuxpan and Tamiahua, the equine seroprevalences for VEEV were as high as 69% and 75%, respectively. Although the sample sizes were small for individual farms (n = 4 or 10) in the municipality of Tamiahua, VEEV antibodies were detected in at least 75% of the sampled bovids and/or equids on the Barra and Tamiahua farms.

### Seroprevalence of dogs for VEEV in the municipality of Minatitlan in the State of Veracruz

In 2008–2009, companion dogs were serosurveyed in areas surrounding Ejido Coachapa in the municipality of Minatitlan in the State of Veracruz. Sera were tested in all cases by both PRNT and HI assays, and there were no differences in the results. All 6 of the dogs tested were seropositive for VEEV with titers ranging from 1∶80–1∶640 by HI and 1∶40–1∶320 by PRNT. The dogs were 6–8 months old, suggesting recent VEEV exposure.

### Seroprevalence of wild-caught rodents for VEEV in the municipality of Minatitlan in the State of Veracruz

In 2008–2009 in the municipality of Minatitlan, 3 species of rodents were trapped in Ejidos Coachapa, in a grassland area near the center of the town (N17°53′25.50″; W94°33′33.30″) and in Ejidos Tacoteno (N18°00′24.62″; W94°30′56.72″). Traps were placed next to swampy pastures and streams of farms where sentinel hamster mortalities and/or positive equine and bovine serology for VEEV had been previously reported. The following rodents were trapped in these locations: *Sigmodon toltecus* (n = 12), *Oryzomys chapmani* (n = 6), and *Rattus rattus* (n = 1). Sera collected from the wild-caught rodents were tested in all cases by both PRNT and HI assays, and the results were consistently either positive or negative for VEEV. The *Rattus rattus* (n = 1) was seronegative for VEEV; however, 4 of the 12 *Sigmodon toltecus* were seropositive for VEEV, 3 of which were from Ejido Tacoteno and 1 from Ejido Coachapa. Five of the 6 *Oryzomys chapmani* collected from Ejido Tacoteno were also seropositive for VEEV, suggesting the possible involvement of these rodents in VEEV circulation in this region as in the Pacific Coast [29].

### Seroprevalence of humans for VEEV in the State of Veracruz

**Figure 3:**
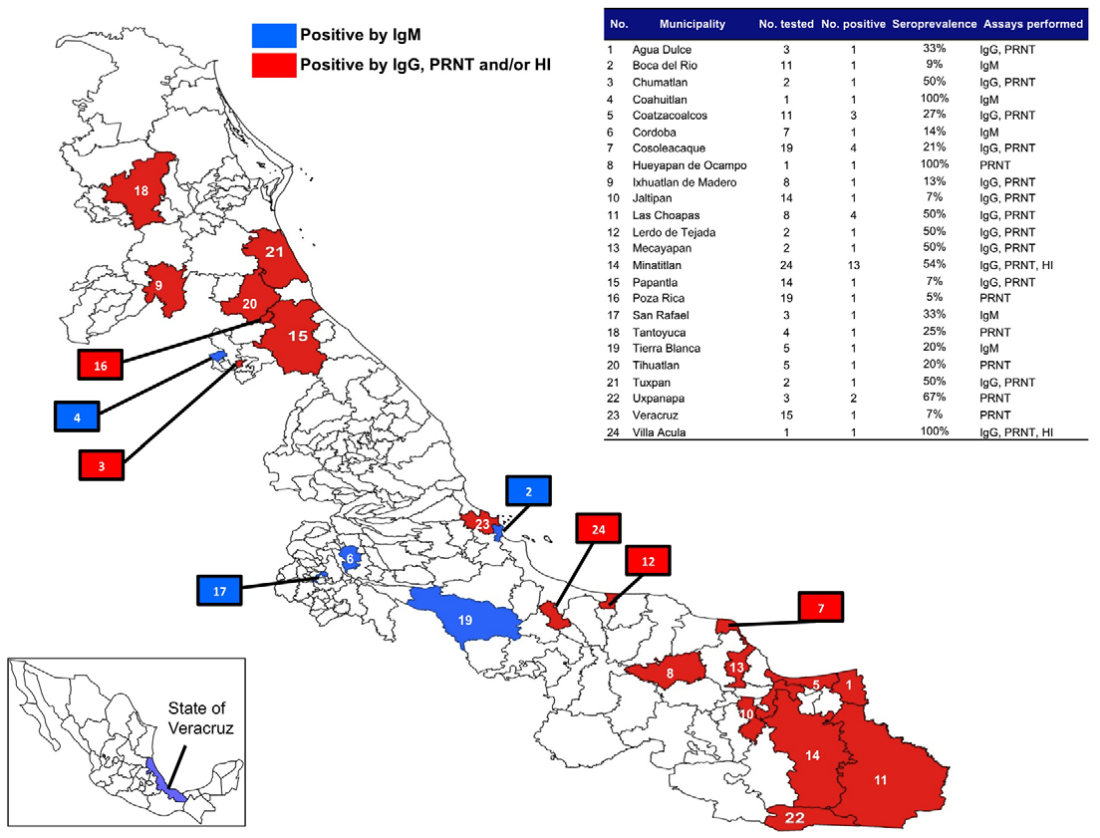
Geographical distribution of humans in municipalities of the State of Veracruz with positive serology by ELISA for IgM/IgG, PRNT, and/or HI for Venezuelan equine encephalitis virus (VEEV), 2008–2009. Red indicates municipalities of the State of Veracruz that were IgM positive for VEEV, and blue indicates municipalities that were positive for VEEV by IgG ELISA, PRNT, and/or HI. Map inset shows the location of the State of Veracruz relative to the other states of Mexico. Table inset provides a location key for each municipality, number of total samples, number of positive samples, seroprevalence (%), and the serology assays that were performed on the samples.

Between October 2008 and April 2009, sera from a total of 234 febrile persons of all ages were obtained from 44 municipalities broadly representing the State of Veracruz, including Minatitlan. Sera were tested in all cases by PRNT, and in most cases, IgG ELISAs. The results were consistently either positive or negative for VEEV. Serum samples from 2 municipalities (Minatitlan, Villa Acula) were also tested by HI assay, and the results were consistent with the PRNT and IgG ELISA results. PRNT- and IgG-negative serum was also tested for IgM antibodies to VEEV. Fig. 3 only shows the seropositive results based on 24 of the 44 municipalities, where all other municipalities had no seropositive results. Based on the results of the ELISA for IgM or IgG, PRNT, and/or HI, 19.2% (45/234) of the total individuals tested were positive for VEEV antibodies. Among these, 5 were IgM-positive, suggesting recent VEEV infection (Table 4). Interestingly, 4 of the 5 IgM positive individuals also showed IgM antibodies to dengue virus (DENV), suggesting the possibility of co-infection. In the Minatitlan region, 13 of the 24 (54%) tested individuals were seropositive for VEEV. This region was also the area where 17 isolates of VEEV were made from sentinel hamsters and mosquitoes during this same time period (2008–2010) (Table 1).

**Table 4:** IgM seroprevalence for Venezuelan equine encephalitis virus (VEEV) in humans located in the State of Veracruz, 2008–2009.

Location^a^	Age (years)	Gender	Ratio of P/N^b^
Coahuitlan	16	Male	3.7
San Rafael	51	Male	4.1
Tierra Blanca	34	Female	3.6
Cordoba	13	Female	4.3
Boca del Rio	42	Female	3.8

a See Fig. 3 for geographic location of these individuals.

b O.D. ratios of sample serum on viral antigen (P)/normal control serum on viral antigen (N); P/N≥3.0, positive; P/N = 2.0–3.0, equivocal; P/N≤2, negative.

### Phylogenetic analyses of viral isolates from the municipality of Minatitlan in the State of Veracruz

**Figure 4:**
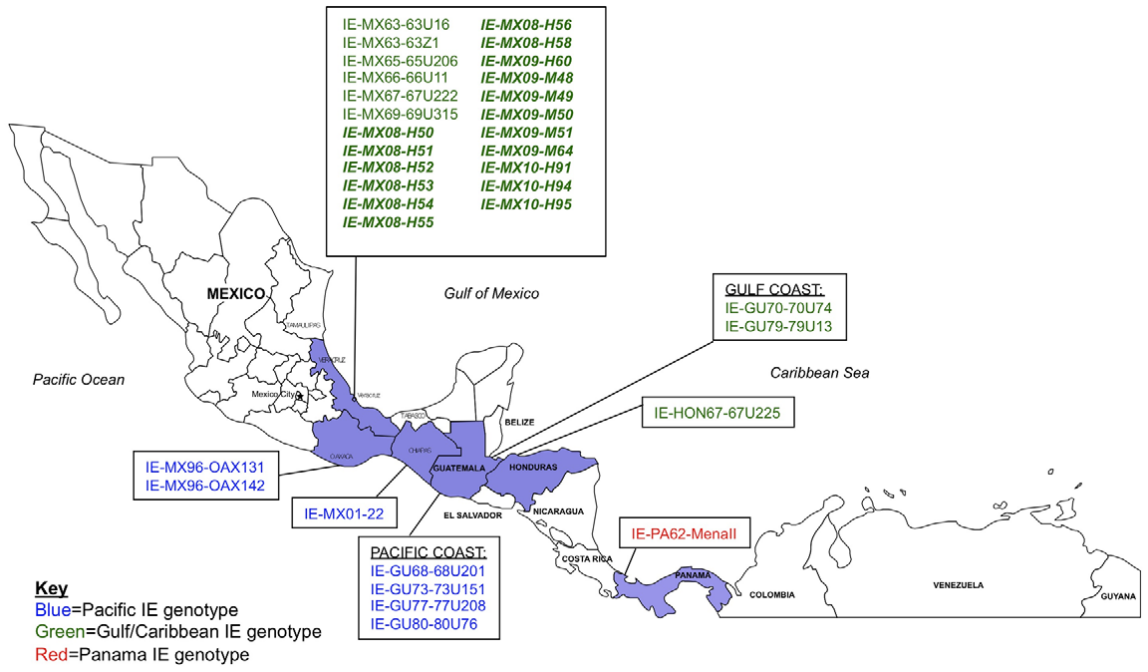
Geographic distribution of subtype IE Venezuelan equine encephalitis virus (VEEV). Based on phylogenetic analyses of these strains, Pacific IE genotypes are represented by blue, Gulf/Caribbean IE genotypes are represented by green, and Panama IE genotypes are represented by red. Recent isolates (2008–2010) are printed in bold.

**Figure 5:**
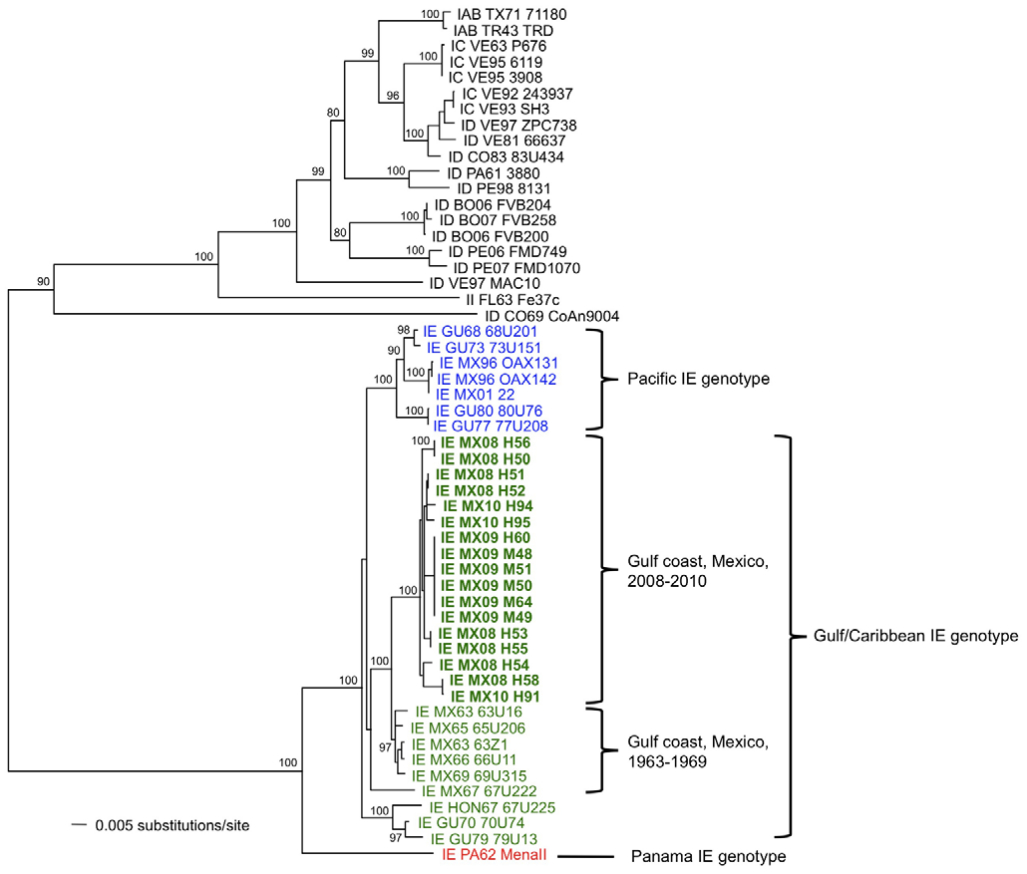
Neighbor joining (NJ) phylogenetic tree of Venezuelan equine encephalitis virus (VEEV) strains based on a 1677 nt fragment, which extends from the 3′ end of the capsid to the 5′ end of E1 within the structural protein genome region. The tree shows the clustering pattern of the sequences obtained from this study as well as representative strains that were available from the GenBank database. VEEV subtype IIIA Mucambo virus was used as the outgroup. Trees generated using maximum parsimony and maximum likelihood (ML) methods had identical topologies. The numbers adjacent to the nodes indicate NJ bootstrap values for 1000 replicates. The scale bar represents the number of nucleotide substitutions per site, and the brackets indicate the genotypes of the subtype IE VEEVs and some geographical/temporal data within the Gulf/Caribbean IE genotype. Sequence names are marked by VEEV subtype, a two-letter abbreviation for the country of origin, a two digit number indicating year of isolation, and the strain name. Pacific IE genotypes are represented by blue, Gulf/Caribbean IE genotypes are represented by green, and Panama IE genotypes are represented by red. Recent isolates (2008–2010) are printed in bold.

Seventeen VEEV isolates were made from sentinel hamsters and mosquitoes collected from the municipality of Minatitlan in 2008–2010. Table 1 provides a listing of the locations, dates of collection, and sources of each isolate, and Fig. 4 shows the geographical distribution of these and previously sequenced isolates of subtype IE VEEV that were used for the phylogenetic analysis. Of the 3 major lineages of subtype IE VEEV, all isolates from the hamsters and mosquitoes clustered within the Gulf/Caribbean genotype but were in a distinct clade from isolates collected in the same regions of Mexico in 1960s (Fig. 5).

The deduced amino acid sequences of the E3 and E2 proteins were also compared across all subtype IE VEEV strains (Table 5). When compared to the Panama and Pacific genotypes, several amino acid differences were distinct for the Gulf/Caribbean IE genotype. Within the Gulf/Caribbean IE genotype, there were no consistent amino acid differences between the 2 temporally distinct isolates from Mexico (1963–69 vs. 2008–10). The amino sequences from 1 sentinel hamster (H60) and 5 mosquito pools (M48, M49, M50, M51, M64), which had been collected from nearby regions in the State of Veracruz in 2009, had a serine→threonine change at position 208 in the E2 protein.

**Table 5:** Deduced E3 and E2 amino acid sequence differences among VEEV subtype IE strains.

Genotype	Virus^a^	E3 residue		E2 residue^b^																			
		17	38	91	117	134	148	150	176	181	197	208	218	224	247	303	313	357	375	377	407	413	414
Panama	IE-PA62-MenaII	***Ser***	Ile	His	Glu	Val	Glu	Pro	Ser	***Thr***	***Leu***	Ser	Ser	***Ala***	***Ala***	Glu	***Asn***	Thr	Ala	Ile	Arg	Ala	Val
Pacific	IE-GU68-68U201	Thr	Ile	His	Glu	***Ala***	Glu	Pro	Ser	Met	Gln	Ser	Ser	Ser	Ser	Glu	His	Thr	Ala	Ile	***Ser***	Ala	Val
	IE-GU73-73U151	Thr	Ile	His	Glu	***Ala***	Glu	Pro	Ser	Met	Gln	Ser	Ser	Ser	Ser	Glu	His	Thr	Ala	Ile	Arg	Ala	Val
	IE-GU77-77U208	Thr	Ile	His	Glu	***Ala***	Glu	Pro	Ser	Met	Gln	Ser	Ser	Ser	Ser	***Asp***	His	Thr	Ala	Ile	Arg	Ala	Val
	IE-GU80-80U76	Thr	Ile	His	Glu	***Ala***	Glu	Pro	Ser	Met	Gln	Ser	Ser	Ser	Ser	***Asp***	His	Thr	Ala	***Leu***	Arg	***Val***	Val
	IE-MX96-OAX131	Thr	Ile	His	***Lys***	***Ala***	Glu	Pro	Ser	Met	Gln	Ser	***Asn***	Ser	Ser	Glu	His	Thr	Ala	Ile	Arg	Ala	Val
	IE-MX96-OAX142	Thr	Ile	His	Glu	***Ala***	Glu	Pro	Ser	Met	Gln	Ser	***Asn***	Ser	Ser	Glu	His	Thr	Ala	Ile	Arg	Ala	Val
	IE-MX01-22	Thr	Ile	His	Glu	***Ala***	Glu	Pro	Ser	Met	Gln	Ser	***Asn***	Ser	Ser	Glu	His	Thr	***Thr***	Ile	Arg	Ala	Val
Gulf/Caribbean (2008–2010)	**IE-MX08-H50**	Thr	Ile	His	Glu	Val	Glu	Pro	Ser	Met	Gln	Ser	Ser	Ser	Ser	Glu	His	***Lys***	Ala	Ile	Arg	Ala	Val
	**IE-MX08-H51**	Thr	Ile	His	Glu	Val	Glu	Pro	Ser	Met	Gln	Ser	Ser	Ser	Ser	Glu	His	Thr	Ala	Ile	Arg	Ala	Val
	**IE-MX08-H52**	Thr	Ile	His	Glu	Val	Glu	Pro	Ser	Met	Gln	Ser	Ser	Ser	Ser	Glu	His	Thr^c^	n/a	n/a	n/a	n/a	n/a
	**IE-MX08-H53**	Thr	Ile	His	Glu	Val	Glu	Pro	Ser	Met	Gln	Ser	Ser	Ser	Ser	Glu	His	Thr^c^	n/a	n/a	n/a	n/a	n/a
	**IE-MX08-H54**	Thr	Ile	His	Glu	Val	***Asp***	Pro	Ser	Met	Gln	Ser	Ser	Ser	Ser	Glu	His	Thr^c^	n/a	n/a	n/a	n/a	n/a
	**IE-MX08-H55**	Thr	Ile	His	Glu	Val	Glu	Pro	Ser	Met	Gln	Ser	Ser	Ser	Ser	Glu	His	Thr^c^	n/a	n/a	n/a	n/a	n/a
	**IE-MX08-H56**	Thr	Ile	His	Glu	Val	Glu	Pro	Ser	Met	Gln	Ser	Ser	Ser	Ser	Glu	His	***Lys***	Ala	Ile	Arg	Ala	Val
	**IE-MX08-H58**	Thr	Ile	His	Glu	Val	Glu	Pro	Ser	Met	Gln	Ser	Ser	Ser	Ser	Glu	His	Thr^c^	n/a	n/a	n/a	n/a	n/a
	**IE-MX09-H60**	Thr	Ile	His	Glu	Val	Glu	Pro	Ser	Met	Gln	***Thr***	Ser	Ser	Ser	Glu	His	Thr	Ala	Ile	Arg	Ala	Val
	**IE-MX09-M48**	Thr	Ile	His	Glu	Val	Glu	Pro	Ser	Met	Gln	***Thr***	Ser	Ser	Ser	Glu	His	Thr^c^	n/a	n/a	n/a	n/a	n/a
	**IE-MX09-M49**	Thr	Ile	His	Glu	Val	Glu	Pro	Ser	Met	Gln	***Thr***	Ser	Ser	Ser	Glu	His	Thr	Ala	Ile	Arg	Ala	Val
	**IE-MX09-M50**	Thr	Ile	His	Glu	Val	Glu	Pro	Ser	Met	Gln	***Thr***	Ser	Ser	Ser	Glu	His	Thr^c^	n/a	n/a	n/a	n/a	n/a
	**IE-MX09-M51**	Thr	Ile	His	Glu	Val	Glu	Pro	Ser	Met	Gln	***Thr***	Ser	Ser	Ser	Glu	His^d^	n/a	n/a	n/a	n/a	n/a	n/a
	**IE-MX09-M64**	Thr	Ile	His	Glu	Val	Glu	Pro	Ser	Met	Gln	***Thr***	Ser	Ser	Ser	Glu	His	Thr	Ala	Ile	Arg	Ala	Val
	**IE-MX10-H91**	Thr	Ile	His	Glu	Val	Glu	Pro	Ser	Met	Gln	Ser	Ser	Ser	Ser	Glu	His	Thr	Ala	Ile	Arg	Ala	Val
	**IE-MX10-H94**	Thr	Ile	His	Glu	Val	Glu	Pro	***Gly***	Met	Gln	Ser	Ser	Ser	Ser	Glu	His	Thr	Ala	Ile	Arg	Ala	Val
	**IE-MX10-H95**	Thr	Ile	His	Glu	Val	Glu	Pro	Ser	Met	Gln	Ser	Ser	Ser	Ser	Glu	His	Thr	Ala	Ile	Arg	Ala	Val
Gulf/Caribbean (1963–1979)	IE-MX63-63U16	Thr	Ile	His	Glu	Val	Glu	Pro	Ser	Met	Gln	Ser	Ser	Ser	Ser	Glu	His	Thr	Ala	Ile	Arg	Ala	Val
	IE-MX63-63Z1	Thr	Ile	His	Glu	Val	Glu	Pro	Ser	Met	Gln	Ser	Ser	Ser	Ser	Glu	His	Thr	Ala	Ile	Arg	Ala	Val
	IE-MX65-65U206	Thr	Ile	His	Glu	Val	Glu	Pro	Ser	Met	Gln	Ser	Ser	Ser	Ser	Glu	His	Thr	Ala	Ile	Arg	Ala	Val
	IE-MX66-66U11	Thr	Ile	His	Glu	Val	Glu	Pro	Ser	Met	Gln	Ser	Ser	Ser	Ser	Glu	His	Thr	Ala	Ile	Arg	Ala	Val
	IE-MX67-67U222	Thr	Ile	His	Glu	Val	Glu	***Leu***	Ser	Met	Gln	Ser	Ser	Ser	Ser	Glu	His	Thr	Ala	Ile	Arg	Ala	***Ile***
	IE-MX69-69U315	Thr	Ile	His	Glu	Val	Glu	Pro	Ser	Met	Gln	Ser	Ser	Ser	Ser	Glu	His	Thr	Ala	Ile	Arg	Ala	Val
	IE-HON67-67U225	Thr	***Val***	His	Glu	Val	Glu	Pro	Ser	Met	Gln	Ser	Ser	Ser	Ser	Glu	His	Thr	Ala	Ile	Arg	Ala	Val
	IE-GU70-70U74	Thr	***Val***	His	Glu	Val	Glu	Pro	Ser	Met	Gln	Ser	Ser	Ser	Ser	Glu	His	Thr	Ala	Ile	Arg	Ala	Val
	IE-GU79-79U13	Thr	***Val***	His	Glu	Val	Glu	Pro	Ser	Met	Gln	Ser	Ser	Ser	Ser	Glu	His	Thr	Ala	Ile	Arg	Ala	Val

a Recent isolates (2008–2010), printed in bold.

b Distinct amino acid differences are underlined and italicized; n/a, not available.

c E2 amino acid sequence ended at aa 369 for H52-55, H58, M48, and M50.

d E2 amino acid sequence ended at aa 352 for M51.

## Discussion

### Serological studies

Almost 50 years since the first studies of VEEV in the State of Veracruz, we determined that this virus continues to circulate in this and other states in the Gulf Coast region and is primarily located in the coastal ecosystems. Based on equine serologic data obtained in 2003–2004, the VEEV endemic region probably extends from the State of Tamaulipas in the north, which borders Texas, to the State of Tabasco in the south. To confirm active transmission of VEEV, more direct studies should be performed that measure virus activity, including performing virus isolations from mosquitoes in these regions. The highest seroprevalence occurred in the State of Veracruz, primarily involving the municipalities of Minatitlan, A. R. Cabada, and Las Choapas in the southern region of the state. Our serological data also demonstrated that VEEV is probably circulating in other southern regions of Mexico, including the municipalities of Tenosique and Balancan in the State of Tabasco, near the Guatemalan border. In the north, we also found seropositive horses in several towns in the State of Tamaulipas, in close proximity to the Texas border. However, it was unclear due to the lack of information regarding vaccination status and travel history whether these horses had been exposed to VEEV naturally in this region. It was, however, confirmed that horses in a more southern location in the State of Tamaulipas (Aldama municipality) were probably exposed to naturally circulating VEEV, since these horses were confirmed unvaccinated and did not have a history of traveling from this site since birth.

Although we obtained no conclusive evidence that regions at the Mexico-U.S. border were enzootic for VEEV, the potential for outbreaks in this region and the ability of the virus to spread into the U.S. remains a concern, as indicated by the involvement of at least 5 Texas counties, primarily Cameron and Hidalgo, during the 1969–1971 epidemic/epizootic [5], [30], [31]. Additionally, it is also known that several epizootic VEEV vectors inhabit the coastal regions of the States of Tamaulipas and Texas, specifically *Ae. taeniorhynchus* and *Ae. sollicitans*. These mosquitoes also can transmit enzootic strains of VEEV [16]. Moreover, the movement of hundreds-of-thousands of people per year across the Mexico-U.S. border is a concern because infected individuals have the potential to become viremic and transmit the virus to biting mosquitoes [7], [32]. In this regard, the states that were surveyed in our study (States of Tamaulipas, Veracruz, and Tabasco) were relevant because they are vital transit points for many people and animals moving within Mexico [33]. According to some reports, approximately 400,000 people annually from at least 50 different nationalities begin their journey from the Mexico/Guatemala border, including the Tenosique-Balancan region of the State of Tabasco, with the ultimate goal of reaching major cities in the U.S. [34]. This journey would take these individuals from the States of Chiapas and Tabasco, Mexico, through the State of Veracruz, and then, to one of the Mexico-U.S. border towns in the State of Tamaulipas [33], [35]. If immigrants cross the border into the U.S. in areas that are also “hot spots” of endemic or epizootic VEEV activity, they could become the source of VEEV transmission [32], [36]–[39].

More than 13.1 million people live in the Gulf Coast region of Mexico, and a large proportion of this population lives in the coastal lowlands of the three states that we studied [40]. However, one missing element in our studies was the lack of a true understanding of the impact of VEEV as a human disease burden in populations living in coastal ecosystems along the Mexican Gulf Coast. The same problem holds true for regions on the Pacific Coast of Mexico, which primarily involve mangrove habitats. Endemic infections due to VEEV are rarely confirmed, because there are other more common etiologies of febrile illness in these regions, such as dengue. Clinically, a mild case of VEE is easily confused with dengue fever and other arboviral diseases, and a confirmatory laboratory diagnosis is rarely pursued due to the cost. The disease burden due to VEEV in the Gulf Coast of Mexico is therefore probably greatly underestimated [41].

Human sera were obtained from a study of suspected dengue infected patients during an outbreak in 2008–2009 in the State of Veracruz, in which the seroprevalence for VEEV was estimated to be 19.2% (45/234). Since these results only confirm prior exposure to VEEV, more work would be needed to verify that the febrile illnesses were due to VEE. Using diagnoses based on virus isolation and serology, Watts et al. [42] reported that VEEV was responsible for at least 3% of febrile illnesses occurring in the Amazon basin of Peru, an area that is also endemic for both dengue and VEE. This trend has also been observed in the Pacific Coast region of Chiapas, Mexico [JGEF and SCW, unpublished data]. According to data from the Mexican Health Ministry (http://www.dgepi.salud.gob.mx/2010/plantilla/intd_boletin.html), a total of 12,531 suspected cases of dengue fever were registered during the outbreak in 2008–2009 in the Gulf Coast States of Tamaulipas (n = 1,722), Veracruz (n = 6,091), and Tabasco (n = 4,718). Therefore, if it is assumed that 3% of these febrile cases were due to VEEV, at least 376 people in this region may have been infected with VEEV.

### Rodent serology

Our data shows that sylvatic rodents of the genera *Sigmodon* and *Oryzomys* were exposed to VEEV, probably during the enzootic circulation of the virus in the regions of Minatitlan that were considered “hotspots” of VEEV activity. The rodent trappings, which were conducted in swampy areas of Minatitlan, reinforce the view that enzootic VEEV circulates in humid tropical habitats. In our study, the seroprevalence in *Oryzomys chapmani* was 83%, and in *Sigmodon toltecus*, the seroprevalence was 33%; this is the first report describing the seroprevalence for VEEV in rodent species that are native to the Gulf Coast region of Mexico. *Sigmodon toltecus* was previously classified by mitochondrial DNA typing as a subspecies of the *Sigmodon hispidus* species group [43], and primarily located in the lowlands of eastern Mexico. *Oryzomys chapmani* (Chapman's rice rat) was recently reassigned to the genus *Handleyomis* in the family *Cricetidae*. This rat, also known as *Handleyomis chapmani*, is found mainly in the highlands of the States of Tamaulipas, Veracruz, and Oaxaca. However, this is the first report of this species in the lowlands of the State of Veracruz [44]–[46].

Our rodent data potentially support the observations from the State of Chiapas on the Pacific Coast that multiple rodent species have the capacity to contribute to the maintenance of VEEV in nature [47]. Based on previous work, sylvatic rodents from the family *Cricetidae*, especially in the genera *Oryzomys* and *Sigmodon*, appear to be involved as reservoir hosts of enzootic VEEV because they are abundant, develop viremia, and exhibit high rates of immunity [3], [5], [47], [48]. However, more extensive studies are clearly indicated to definitely identify which reservoir hosts have the ability to transmit VEEV to mosquito vectors in the Gulf Coast region. Our data also does not rule out the possibility that other rodent species within communities along the Gulf Coast region play a role in the maintenance of VEEV. Future surveillance studies should include larger geographic regions and more rodent (and other mammalian) species.

### Phylogenetic analysis

Based on our phylogenetic analyses, all VEEV isolates from the municipality of Minatitlan fall within the Gulf/Caribbean IE genotype. The results indicate that the VEEV strains currently circulating in the State of Veracruz probably evolved from those circulating in this area nearly 50 years ago. These results also suggest that there have been no introductions of the VEEV Pacific genotype (or the Panama genotype) into this region of the Gulf Coast. Sequence analysis did not show a Ser→Arg change at amino acid position 218 of the envelope glycoprotein E2, which has been previously implicated in the adaptation of subtype IE VEEV to the epizootic mosquito vector, *Ae. taeniorhynchus* [16]. Future phylogenetic studies should include isolates from all regions of the Gulf Coast to determine whether the Pacific Coast lineage with epizootic potential has been introduced into other areas of Mexico.

### Conclusion

Overall, our results indicate that the epidemiological pattern of VEEV in Mexico may be changing. According to the 2010 census, more than 75% of Mexican people reside in urban centers [40]. Our study showed that at least one major urban region (Minatitlan in the State of Veracruz) has active enzootic VEEV transmission. Thus, an epidemic strain of VEEV has the potential to arise from circulating endemic strains, which may be easily misdiagnosed for another febrile-causing disease if appropriate diagnostic assays are not routinely performed. The movement of epidemics by viremic individuals is another concern, particularly in the Gulf Coast region of Mexico, which could threaten previously unaffected areas of Mexico and even the U.S. These scenarios underscore an urgent need for public health policy makers of Mexico and the U.S. as well as the scientific community to continue to develop strategies for the prevention and control of VEE.

## Supporting Information

Table S1 **Primers used for genetic amplification and sequencing reactions.**
(DOC)
