# Supplementary material for: Venezuelan Equine Encephalitis Virus Activity in the Gulf Coast Region of Mexico, 2003–2010
Source: PLoS Negl Trop Dis. 2012 Nov 1;6(11):e1875. doi: 10.1371/journal.pntd.0001875 (PMC3486887; doi:10.1371/journal.pntd.0001875)
Supplement: Table S1 — Primers used for genetic amplification and sequencing reactions. (DOC) [file pntd.0001875.s001.doc]

Table S1. Primers used for genetic amplification and sequencing reactions

| Primer name^a^ | Nucleotide sequence (5’ to 3’) |
| --- | --- |
| 7894(+)^b^ | GTCAATAGGAAGCCAGGGAAGAG |
| 8642(-) | GCACACCTGATGCACCTG |
| 8423(+) | TAGCCAATGTSACGTTCCC |
| 9783(-) | ATGCTGCGATGGATGTCGTCAC |
| 9162(+) | AGTGGAATGCGAATGTGGTGGC |
| 10395(-)^b^ | TGAGGCTGTATGTGCTTTGTATGC |

^a^Numbers correspond to the position of the 5’ nucleotide of the primer based on the full-length genome of VEEV strain 68U201.

^b^Primers used for RT-PCR; these and all other listed primers were used for sequencing.
